# Variable Rate Point Cloud Geometry Compression Method

**DOI:** 10.3390/s23125474

**Published:** 2023-06-09

**Authors:** Lehui Zhuang, Jin Tian, Yujin Zhang, Zhijun Fang

**Affiliations:** 1 The School of Electronic and Electrical Engineering, Shanghai University of Engineering Science, Shanghai 201620, China; m020220113@sues.edu.cn (L.Z.); jintian0120@foxmail.com (J.T.); jyzhang@sues.edu.cn (Y.Z.); 2 The School of of Computer Science and Technology, Donghua University, Shanghai 201620, China

**Keywords:** point cloud compression, variable bit rate, contrastive learning

## Abstract

With the development of 3D sensors technology, 3D point cloud is widely used in industrial scenes due to their high accuracy, which promotes the development of point cloud compression technology. Learned point cloud compression has attracted much attention for its excellent rate distortion performance. However, there is a one-to-one correspondence between the model and the compression rate in these methods. To achieve compression at different rates, a large number of models need to be trained, which increases the training time and storage space. To address this problem, a variable rate point cloud compression method is proposed, which enables the adjustment of the compression rate by the hyperparameter in a single model. To address the narrow rate range problem that occurs when the traditional rate distortion loss is jointly optimized for variable rate models, a rate expansion method based on contrastive learning is proposed to expands the bit rate range of the model. To improve the visualization effect of the reconstructed point cloud, a boundary learning method is introduced to improve the classification ability of the boundary points through boundary optimization and enhance the overall model performance. The experimental results show that the proposed method achieves variable rate compression with a large bit rate range while ensuring the model performance. The proposed method outperforms G-PCC, achieving more than 70% BD-Rate against G-PCC, and performs about, as well as the learned methods at high bit rates.

## 1. Introduction

The increasing availability of 3D sensors has driven a wave of innovation of immersive devices, such as Augmented and Virtual Reality Production. In 2020, Apple successfully created a more realistic augmented reality experience by bringing point cloud to mobile devices [1]. Point cloud have achieved significant success in some emerging industries for its high resolution and high fidelity. However, its huge data volume has brought great inconvenience to the transmission and storage. Data compression methods help solve the problem of excessive data volume in its storage and transportation [2]. Therefore, point cloud compression (PCC) technology has become one of the urgent challenges to be broken in 3D sensor applications.

Point cloud is a collection of a large number of discrete points, which holds information about the surface of an object in the form of 3D data. It is widely used in industrial scenes due to its high accuracy and high resolution. However, due to its extremely large data volume, which puts great pressure on storage and transmission, efficient PCC algorithms are urgently needed.

Deep learning has achieved excellent results in many computer vision tasks. With the revolutionary progress in deep learning, learned PCC has attracted much interest. In particular, non-linear transform coding designed by deep neural networks has achieved impressive rate distortion, and even some algorithms outperform classical PCC codecs designed and optimized by domain experts, such as the G-PCC [3] proposed by MPEG.

Lossy point cloud compression takes advantage of smaller storage space and transmission costs at the cost of reduced reconstructed point cloud quality. These learned compression methods [4,5,6,7] utilize auto-encoders or generative models to learn compact feature representations. An encoder transforms the point cloud into a latent feature representation, and a decoder converts the latent features back to the point cloud. The transformation is designed to obtain the latent representation with the smallest entropy to reduce the compression rate for a given distortion level. The entropy of latent representation is usually difficult to calculate directly. Hence, the rate–distortion (R-D) trade-off is optimized by minimizing an entropy estimate of the latent representation of point cloud. Some accurate entropy estimation models [8,9,10] have been developed to improve compression efficiency.

Most of the learned PCC methods optimized their networks by minimizing the sum of the R-D pairs using the method of Lagrange multipliers. The Lagrange multiplier λ is a hyperparameter to train a network for a desired compression rate, which indicates that separate networks for different compression rate are needed to be trained and deployed. However, it is impractical to operate with fine resolution over a wide range of R-D curves. Therefore, a variable rate point cloud compression model is of great interest.

The variable-rate compression model trains only one network, and the compression rate can be flexibly adjusted by the hyper-parameter, which saves a lot of storage space. At the same time, since the bit rate change of the variable rate model is continuous, the compression rate can be adjusted according to the network bandwidth, which effectively improves the transmission efficiency.

Despite the excellent performance achieved by learned PCC methods, variable rate PCC still has many difficulties. First, most of the currently available variable rate models are in the field of image compression, while only a small amount of work has been done in learned PCC. It is urgent to explore the variable rate PCC framework. Moreover, the sum of R-D pairs for model optimization cannot meet the variable rate model requirements in the training.

In this paper, we have proposed a variable rate point cloud geometry compression framework, named VRPCGC. In particular, we have proposed the modulated network, which takes the Lagrange multiplier as an input and produces the weight of latent representation. The compression rate depends on the input value. Meanwhile, we suggest keeping changing the Lagrange multiplier during training. It should be noted that the Lagrange multiplier λ are set to discrete values during training, but are continuous in the test, which ensures that continuous compression rates are achieved. Meanwhile, we have proposed a contrastive learning to increases the range of compression rates of the model. Specifically, models with different hyperparameters are considered as negative pairs to distance their latent representation during training, which pulls apart the rate distribution of the model.

Furthermore, boundary learning is introduced to improve the visualization of reconstructed point clouds. Tang et al. [11] proposed boundary learning in point cloud segmentation to improve the classification ability of point cloud boundaries. In this paper, we use the boundary information of point cloud to increase the accuracy of classifier on edge regions and improve the texture details of reconstructed point clouds, which give the reconstructed point clouds better visualization quality.

In summary, the main contributions of this paper are:

1. We have proposed a variable rate point clouds geometry compression framework based on modulation network. We analyze the impact of different modulation networks on the model performance, and the experimental results show that the convolution neural network achieves better performance.
2. We have introduced the contrastive learning to increase the bit rate range of the model, which solves the bit rate concentration problem brought by the traditional rate distortion optimization.
3. We have proposed the boundary learning for the point cloud reconstruction, which focuses on the boundary points to ensure the visualization quality of the point cloud.

## 2. Related Work

Related researches of this work can be classified into learned point cloud compression and variable rate compression.

### 2.1. Learned Point Cloud Compression

Deep learning has achieved remarkable results in data processing. Zhao et al. [12,13] introduced deep learning in federal learning and achieved remarkable results. Jin et al. [14] proposed using deep transfer learning from face recognition to perform the computer-aided facial diagnosis on various diseases. Liu et al. [15] proposed a generative adversarial network for point cloud upsampling to generate point clouds with the better visual quality.

The learned compression approach has gained much attention due to its excellent rate distortion performance. The compression process could be divided into lossy and lossless compression according to whether there is a loss in the quality of the point cloud. Lossless compression methods [7,16] use neural networks to estimate the probability of point cloud occupancy, and then the estimated probability is losslessly compressed by an arithmetic encoder. However, it is difficult to achieve high compression rates in this way. More studies [17,18] want to achieve higher compression ratios with less distortion. A tighter point cloud representation is obtained by downsampling, and then the point cloud is reconstructed by upsampling at the decoder.

Currently, deep learning is applied to the octree domain [19,20], voxel domain [21,22,23,24] and point domain [25] of PCC. Huang et al. [19] have proposed an octree structured conditional entropy model to model the probability of octree symbols and encode the octree symbols into a compact bit stream. However, this approach leads to an exponential decrease in the number of point clouds as the depth of the octree decreases as well as the block effect phenomenon. Que et al. have proposed VoxelContext-Net, which achieves better entropy estimation by using neighboring points of parent nodes as contextual information.

Quach et al. [21], Wang et al. [22] and Guarda et al. [23] have proposed a 3D convolution-based PCC. They have transformed the point cloud reconstruction task into a binary classification task, and optimized the geometric information distortion of the point cloud according to the binary classification loss. Wang et al. [24] have made further optimization of the voxel-based PCC method and proposed a sparse convolution-based PCC framework, which greatly reduces the computational. Nguyen et al. [26] have attempted to mix octree and voxel-based coding, which partition the point cloud into multi-resolution voxel blocks. You et al. [25] have proposed a direct way to deal with points, they divide the point cloud into multiple blocks, encode each block independently, and recombine all patches into a complete point cloud in the decoding process.

### 2.2. Variable Rate Compression

Most of the currently available variable rate models are in the field of image compression. Toderici et al. [27] is the first to propose a variable compression approach based on LSTM. The learned variable rate model achieves the effect of changing the compression bit rate by deflating the features. Cui et al. [28] and Guo et al. [29] have proposed the gain matrix to recover the features at the decoder. Gupta et al. [30] have proposed to weigh the importance of the features at different locations by the importance distribution map. However, the corresponding importance correlation maps are difficult to obtain in practical applications. Choi et al. [31] have designed a conditional autoencoder structure to adjust the feature weights between different channels and introduced a hyperparameter into the training process of rate distortion optimization.

In the field of PCC, Kathariya et al. [32] have proposed a variable rate compression method based on tree structure. However, this method only deals with point clouds represented by the tree structure and cannot be applied to point clouds of other structures. In order to avoid the errors caused by voxelization, Muzaddid et al. [33] have proposed a direct treatment of point cloud model structures, and they have proposed a weighted entropy loss and inference strategy to achieve the variable rate compression task.

Huo et al. [34] have proposed variable-rate point cloud attribute compression, which adjusts the weights of different channels of point cloud features through multiple scale networks. This is the only learned variable rate point cloud compression model as we know. However, they only address attribute compression of point clouds and does not apply to the domain of geometry compression of point clouds. Moreover, they do not designed the corresponding objective optimization function according to the variable rate model, which often produces no ideal model performance.

## 3. Proposed Method

This section provides a detailed description of the proposed VRPCGC. We introduce the three aspects of the system, namely, the overall framework, the modulation network, and the improved rate-distortion optimization.

### 3.1. Overall Framework

**Figure 1:**
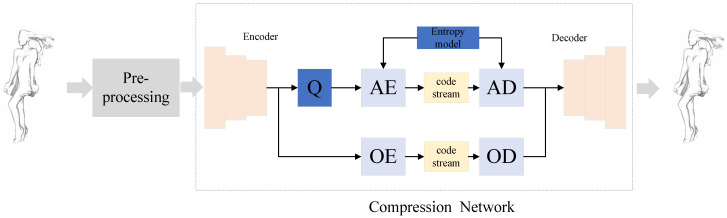
Learned point cloud compression framework, which is composed of pre-processing and compression network. “Q” stands for “Quantization”. “AE” and “AD” are Arithmetic Encoder and Decoder, respectively. “OE” and “OD” are Octree Encoder and Decoder, respectively.

The proposed framework is presented in Figure 1. The two main components are pre-processing and compression network. The pre-processing converts the point cloud into a data format suitable for compression network processing, which transforms the point cloud into a volume model through point cloud voxelization. The volume model represents the point cloud geometry information by occupancy state, using “1” to indicate that the point is occupied and “0” to indicate that it is not occupied. After the voxelization, the point cloud is encoded and decoded using a compression network.

The compression network is based on autoencoder structure, where the autoencoder takes the input information as the learning target and becomes a high-dimensional representation of the data. It includes encoder network, quantization and entropy model and decoder network, which can be expressed by Equation (1)

(1) $$y=E\left(x;\theta \right),\;\;\;\;{\hat{F}}_{y}=Q\left(F_{y}\right),\;\;\;\;\hat{x}=D\left(\hat{y};\delta \right)$$

where E(·), Q(·) and D(·) represent encoding, quantification, and decoding function, θ and δ are the parameters of encoder network and decoder network, respectively. It is important to note that the *y* is composed of $G_{y}$ and $F_{y}$, which are geometric coordinates and latent feature of point cloud, respectively. The former is losslessly compressed by the octree codec, while the latter is losslessly compressed by an arithmetic encoder. So only $F_{y}$ is quantified. In practice, we usually approximate this process by adding uniform noise as follows:

(2) $$\hat{y}=y+\mu$$

where μ is uniform noise ranging from −0.5 to 0.5. In addition to adding uniform noise, gradient back-propagation can also be achieved by soft quantization [8] or skipping quantization layers to ensure the training of the model.

**Figure 2:**
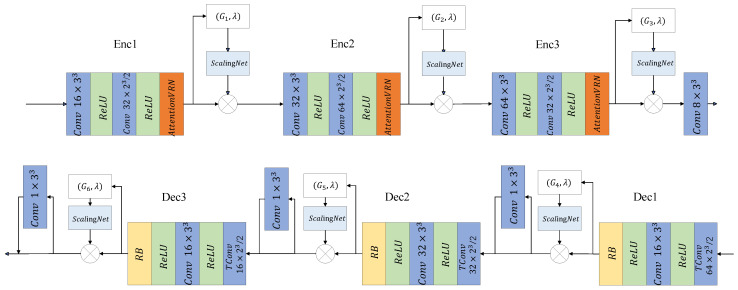
Detailed architecture of the encoder and decoder. “ReLU” stands for the Rectified Linear Unit. “Attention-VRN” is the Attention Voxception ResNet for feature aggregation.

Figure 2 (Top) displays the encoder network structure, which mainly includes three parts, down-sampling, feature enhancement, and modulation network. The down-sampling operation is implemented by sparse convolution with a stride size of 2, which reduce the spatial scale of point cloud and obtain a denser point cloud. The attention mechanism is often used to improve the network performance [35,36]. The AttentionVRN is designed to capture the effective feature information using the attention mechanism. The ScalingNets adjust the current features according to hyperparameter to achieve control of the bit rate.

Figure 2 (Bottom) shows the decoder network structure, which mainly includes upsampling, feature enhancement, classifier, and scaling networks. The upsampling operation is implemented by transpose convolution with step 2 to recover the scale of the point cloud. A simpler structure of residual block (RB) is used for feature enhancement. Each feature enhancement module is followed by a scale network.

The encoder network obtains a compact latent representation by three downsamplings, which contains the contextual information of the point cloud. Scaling networks are embedded in the model to control the compression rate of the model. The input to the scaling network contains not only the Lagrange multiplier but also the geometric information of the current point cloud, which gives the model the ability to focus on the spatial characteristics.

**Figure 3:**
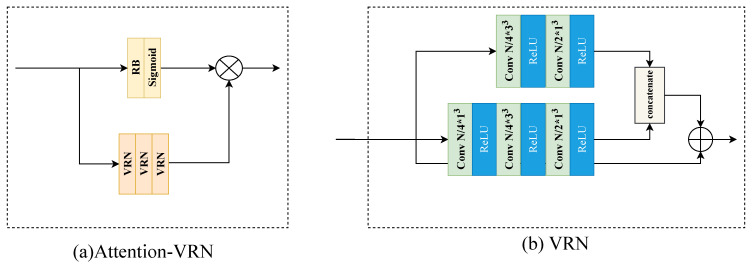
Architecture of AttentionVRN and submodules.

The AttentionVRN structure is shown in Figure 3, where Figure 3a displays the overall structure of the AttentionVRN, and Figure 3b displays the VRN [37] structure. The AttentionVRN contains two branches, the main branch uses three consecutive VRNs to extract features, and the mask branch is computed by a ResBlock. A Sigmoid activation is applied to obtain the joint spatial channel attention mask M. The calculation process is shown in Equation (3)

(3) $$M=Sigmoid(RB\left(F_{in}\right))$$

where $F_{in}$ is the input feature and *M* is the generated attention mask.

After downsampling, the geometric information of point cloud is compressed separately from the latent features by the traditional coders. The geometric coordinates of the point cloud are losslessly encoded by an octree encoder while the latent features are first quantized and then lossy encoded using an arithmetic encoder, which is assisted by a conditional encoder to improve the encoding efficiency.

The point cloud is reconstructed by upsampling in the decoder, and the details of point cloud are recovered using classification function. The boundary learning is proposed for the point cloud reconstruction. The points in the point cloud are classified in the spatial structure, and the edge point determination method is proposed to focus on the edge points and their neighborhood information. A boundary loss is proposed to enhance the classification ability for boundary points. The decoder network is also embedded with scale networks to adjust the decoding features when the model compression rate changes and reduce the distortion of the point cloud.

### 3.2. Variable Rate Point Cloud Geometry Compression

**Figure 4:**
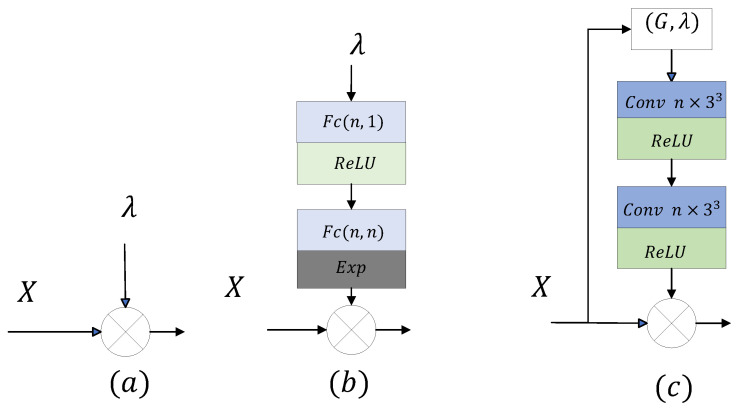
Different feature modulation methods (**a**–**c**).

We have proposed a variable rate point cloud geometry compression via ScalingNets, which modulate the latent features of different layers in the encoder and the decoder. As shown in Figure 4a, a simple way to modulate the features is to multiply the hyperparameter directly with the current features, which can be expressed as Equation (4):

(4) $$X^{\prime }=\lambda X\;$$

where *X* is the input tensor. However, such a regulation is too simple to achieve the ideal results.

Figure 4b displays a common scaling network modulated in the channel dimension, consisting of two full connection networks. It means multiplying the feature map and the modulating/demodulating tensor in a channel-wise production manner. Civen a feature map *X*, the output map of scaling network can be calculated as

(5) m(λ)=exp(w2ReLU(w1λ))

where $w_{1}$ and $w_{2}$ are the parameters of the two fully connected layers, respectively. Then a channel-wise production is preformed:

(6) $$X^{\prime }=m\left(\lambda \right)X\;$$

However, it is difficult to achieve fine modulation and to effectively control tensor with few feature channels. The small number of feature channels in the geometric information compression process of point clouds makes this approach unsuitable to apply.

Figure 4c displays the structure of the convolution-based scaling network proposed in this paper. The input of this network contains not only the hyperparameter, but also the geometric information of the input tensor. Specifically, the hyperparameters are used as the attribute information of the current point cloud to form a new sparse tensor. The network consists of two sparse convolutions, each of which uses a Relu activation function. The output tensor is the same size as the input and is multiplied by the corresponding elements in the input tensor. This scaling network not only achieves more accurate modulation, but also learns the spatial information of the point cloud due to the input geometric information.

The variable rate point cloud compression model based on the above three scaling networks are named VRPCGC(I), VRPCGC(II), and VRPCGC(III).

**Figure 5:**
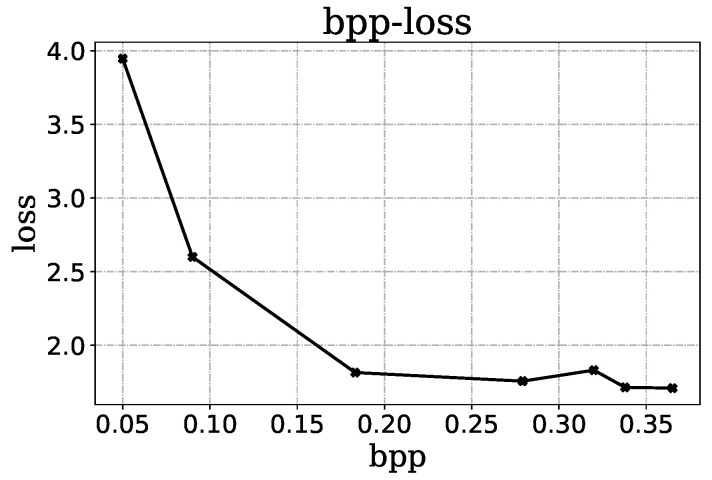
Rate-loss curve during the training.

The sum of the R-D pairs using the method of Lagrange multipliers is usually optimized for a desired performance. However, it causes the compression rate to be concentrated in the high bit rate region. As shown in Figure 5, the overall loss of the model is much lower at the high rates than at the low rates, which makes it difficult to obtain a low rate model.

Contrastive learning maps the data to the projection space, and then closes the distance between positive samples, while pulling the distance between negative samples. Inspired by the contrastive learning, the models corresponding to different Lagrange multipliers are expected to be less similar to each other in order to pull apart the rate distribution of the models. Therefore, it is beneficial to obtain a model with a wider compression rate range by treating the models corresponding to different Lagrange multipliers as negative samples to enlarge their distance. Therefore, a rate similarity penalty is proposed, which is calculated as Equation (5)

(7) $$L_{con}=1/\sum \exp(Sim\left(R,R_{former}\right)/\tau )$$

where *R* is the bit rate of the current model, $R_{former}$ is the bit rate of the previous model, τ is the temperature coefficient, which controls speed of change. Sim(·) is the similarity between the two bit rates, which is calculated as Equation (6):

(8) $$Sim\left(R,R_{former}\right)=\left\{R/R_{former},R<R_{former}R_{former}/R,R\geq R_{former}\right\}$$

### 3.3. Boundary Learning

Most of the existing point cloud processing methods ignore the segmentation of the scene boundaries. Little attention has been paid to the boundaries of 3D point clouds, while accurate segmentation of scene boundaries is very important [11]. In image segmentation, accurate boundary segmentation is the key to produce high-fidelity masks. Second, incorrect boundary segmentation may cause great harm for some objects with few occupied points, which can have an impact on many downstream tasks.

The accurate segmentation of boundary regions affects the reconstruction quality of point cloud in PCC. The boundary region represents some detailed texture points. Without accurate segmentation, point cloud visualization will be worse. Due to the lack of sufficient points in the boundary area, it is difficult to extract effective information. Therefore, a boundary learning for PCC is proposed.

**Figure 6:**
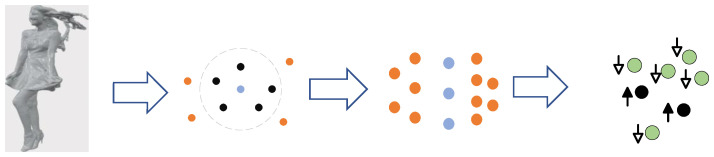
The framework of boundary learning, which is composed of three parts: neighborhood determination, boundary points screening, and boundary points optimization.

As shown in Figure 6, for each point of the ground-truths data, all points that are less than *r* away are considered as its neighborhood. Then, a point is considered as a boundary point when the number of points in its neighborhood is less than ε. For the voxelized point cloud, the radius of the neighborhood is set to 2 to avoid the huge computation due to oversized settings.

For a given point cloud, the boundary points are filtered out first. The classification accuracy of them is considered more important. A boundary learning (BL) loss is proposed to enhance the focus on boundary points. The set of all boundary points and their neighbors in the reconstructed point cloud is counted and denoted as N. Then the Binary Cross Entropy (BCE) loss is used as the distortion in training, i.e.,

(9) $$L_{BL}=\frac{1}{N}\sum_{x_{i}\in N}-[l_{i}\log\left(p_{i}\right)+\left(1-l_{i}\right)\log\left(1-p_{i}\right)]$$

where $p_{i}$ is the occupancy probability estimate of the points in the neighborhood, $l_{i}$ is the true label, and *N* is the number of all points in the domain. The boundary loss reinforces the classification of boundary points and increases the loss weight of the boundary region, whic brings greater attention to the boundary.

### 3.4. Loss Function

In our model, a total rate consumption comes from the ${\hat{F}}_{y}$, and rate approximation can be written as:

(10) $$R=E_{{\hat{F}}_{y}∼p_{{\hat{F}}_{y}}}\left[-\log_{2}q_{\hat{y}}\left({\hat{F}}_{y}\right)\right]$$

The details of the point cloud are recovered by binary classification. The BCE loss is used as the distortion of each up-sampling in training, i.e.,

(11) $$D_{k}=\frac{1}{N}\sum_{i}-[l_{i}\log\left(p_{i}\right)+\left(1-l_{i}\right)\log\left(1-p_{i}\right)]$$

where $p_{i}$ is the occupancy label of the current voxel, $p_{i}$ is the voxel occupancy probability and *N* represents the number of generated points. Considering that the importance of each up-sampling is not the same, different weights should be performed, i.e.,

(12) $$D=\sum_{k}\delta_{k}D_{k}$$

where $\delta_{k}$ denotes the weights, set to (1, 2, 4). In our end-to-end learning framework, we define the loss function considering all the above losses, so as to maximize the overall performance, i.e.,

(13) $$L=R_{{\hat{F}}_{Y}}+\lambda D+\left(1-0.95\lambda \right)L_{\mathrm{con}}+2L_{BL}$$

Since the calculation of the additional loss increases the distortion of reconstructed point cloud, it is necessary to control the weight of $L_{\mathrm{con}}$. When the λ becomes larger, the weight of $L_{\mathrm{con}}$ becomes smaller, which ensures the performance of the model at high bit rates.

## 4. Results and Discussion

### 4.1. Implementation Detail

#### 4.1.1. Dataset

Our model was trained on the well-known 3D shape dataset ShapeNet [38]. We randomly sampled these CAD models to obtain point clouds, with the number of points in each point cloud being random. Meanwhile, a random rotation was performed to increase the diversity of the data. Each coordinate dimension of the point cloud is 7-bit accuracy.

In total, eight dense point clouds were selected for the test, which is from 8i Voxelized Full Bodies (8iVFB) [39], and Owlii dynamic human mesh (Owlii) [40]. These point clouds vary in size and structure. We list some of the information of these point clouds in Table 1. They are used in the MPEG Common Test Conditions (CTC) [41] for compression task exploration.

#### 4.1.2. Training Strategy

The loss function for training is shown in Equation (13). The temperature coefficient τ is set to 2. The model is randomly initialized at the beginning. A model with a high bit rate range is trained while the λ is set to (0.9, 1), which is used as an initialized model for training lower bit rates. The lower bound of λ is continuously decreasing until 0 to obtain a model with a larger bit rate range. In addition, we use the Adam optimizer with the learning rate dynamically declining from $8\times 10^{-4}$ to $1\times 10^{-5}$. The batch size is set to 16.

### 4.2. Performance Evaluation

We have compared the VRPCGC(III) with other PCGC methods, including traditional algorithms and learning-based methods, while the performance of VRPCGC(I) and VRPCGC(II) are analyzed in the ablation experiments. Traditional compression algorithms include G-PCC [3] and V-PCC [42]. G-PCC contains two model representation methods: the octree model and the trisoup model, which are referred to as G-PCC (octree) and G-PCC (trisoup). Learned methods include Learned-PCGC [22] and PCGCv2 [24]. To achieve a fair comparison, the coding parameters were set according to the MPEG PCC General Test Conditions [41] while enforcing the similar bit rate ranges for the all methods.

In this paper, objective performance is evaluated using the BD-Rate, using D1 (p2point) PSNR and D2 (p2plane) PSNR as the distortion matrix and bits per input point (bpp) as the bit rate. The results are shown in Table 2. The PSNR is calculated via the mean squared error (MSE) [43]. Our method outperforms the G-PCC and Learned PCGC by a significant margin, which obtains −83.06% D1 BD-Rate gains and −80.80% D2 BD-Rate gains on average compared to G-PCC (octree), −64.49% D1 BD-Rate gains and −76.66% D2 BD-Rate gains on average compared to G-PCC (trisoup), −16.91% D1 BD-Rate gains and −24.53% D2 BD-Rate gains on average compared to Learn-PCGC. Our method achieves a comparable performance performance with V-PCC. Results have shown that the average +35.60% D1 BD-Rate gains and +6.37% D2 BD-Rate gains are captured. However, the encoding time for V-PCC is too long because it requires chunking and projection of the point cloud. Furthermore, the visualization quality of its decoded point cloud is poor due to its decomposition of the point cloud into multiple chunks. V-PCC performs better on the Owill set. This is because our method takes into account more changes of bit rate, regardless of not adapt well to changes in the data precision. Compared with PCGCv2, our method has some performance gap with it, which is an unavoidable problem for variable rate PCC methods. However, these learned PCC algorithms require training a large number of models to achieve multiple bit rate compression, e.g., the PCGCv2 have trained seven models while our method trained only one model. What is more, our method is able to operate with fine resolution over a wide bit rate range.

**Figure 7:**
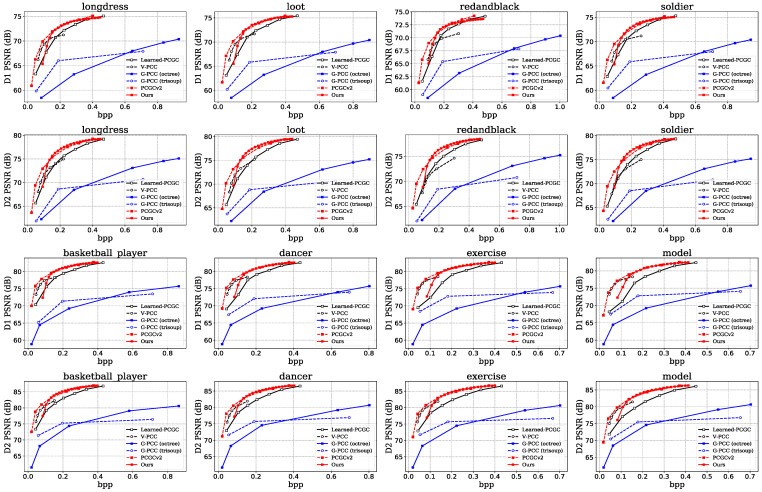
Rate distortion curves plotted on different test samples.

Illustrative Rate-distortion curves are presented in Figure 7. The performance of our method has surpassed most algorithms in the high bit rates, and is close to or even surpasses the best overall performance of PCGCv2. At low bit rates. The performance of our method decreases and is pulled apart by the V-PCC and PCGCv2, but still has a significant advantage over the G-PCC. The performance degradation at low bit rates, causing that the overall performance is not optimal. For variable rate compression models, a certain performance degradation is acceptable under the premise of ensuring the bit rate range of the model.

**Figure 8:**
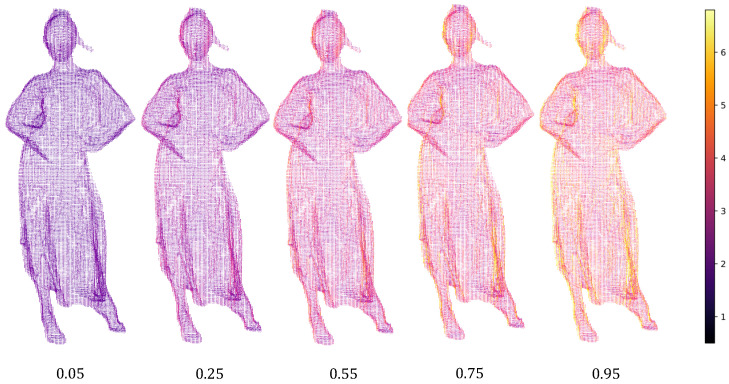
The visualization of latent features of ScalingNet corresponding to different λ.

To further analyze the variable rate model, we have visualized the latent features of the ScalingNet. Figure 8 shows the visualization of one of the layers of features of the second ScalingNet of the encoder at different λ. The scale network output value becomes larger as λ, which amplifies the latent features and the increases the entropy latent features.

It can also be seen that the features within a feature map are not equal everywhere. If the brightness of a feature map is uniform, the ScalingNets scale a tensor directly, which is difficult to achieve precise feature modulation to accommodate bit rate changing, causing the poor performance when λ is small. Our method have achieved a better performance for that the ScalingNets learns the geometric information of the point cloud.

## 5. Ablation Experiments

We further extend our studies by examining various aspects of our VRPCGCC, including the scaling networks, contrastive learning, and the boundary learning, to demonstrate the robust and reliable performance of our method.

### 5.1. Scaling Networks

We have analyzed the performance of the scaling networks which modulate the features in different dimensions. The VRPCGC(I), VRPCGC(II), and VRPCGC(III) are trained for the same number of rounds under the same settings.

**Figure 9:**
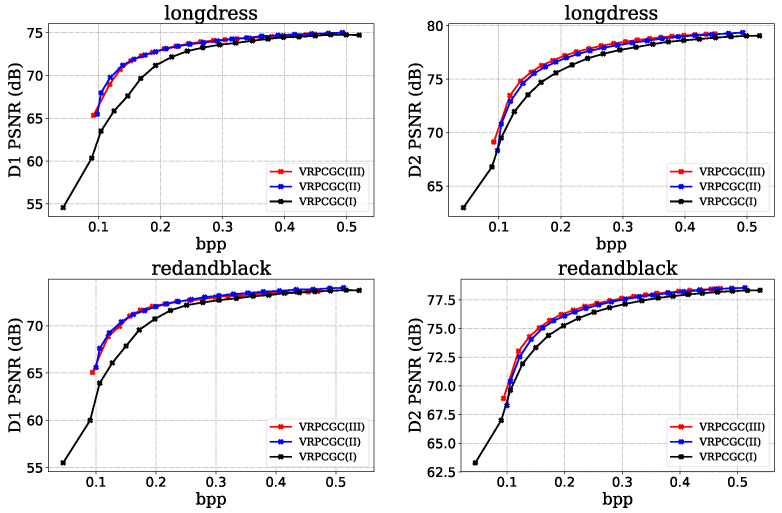
Rate distortion curves based on different ScalingNets.

Figure 9 shows the rate distortion curves of the VRPCGC based on different scaling networks. VRPCGC(I) achieves the largest range of bit rates for that the hyperparameter is able to affect the latent features directly. However, its overall performance is the worst because such a coarse control cannot guarantee the reconstruction quality of the model when the bit rate changes. VRPCGC(II) achieves a relatively good result in D1 PSNR, but does not perform ideally enough in the D2 PSNR metric. Moreover, it is difficult to adapt to low bit rates, and its performance drops sharply at low bit rates. VRPCGC(III) achieves the optimal rate distortion performance because the convolutional layer has powerful ability of feature processing to finely modulate the features and better adapt to the change of bit rate.

For the scaling network, the fine scaling network ensures that model can precisely modulate the feature structure to minimize the distortion when a desired bit rate is changing. The convolution-based scaling network can focus on more important regions based on point cloud geometry information to achieve more effective modulation.

### 5.2. Contrastive Learning

Contrastive learning is proposed to extend the range of bit rates of the model. We aimed to analyze the impact of contrastive learning on model performance.

**Figure 10:**
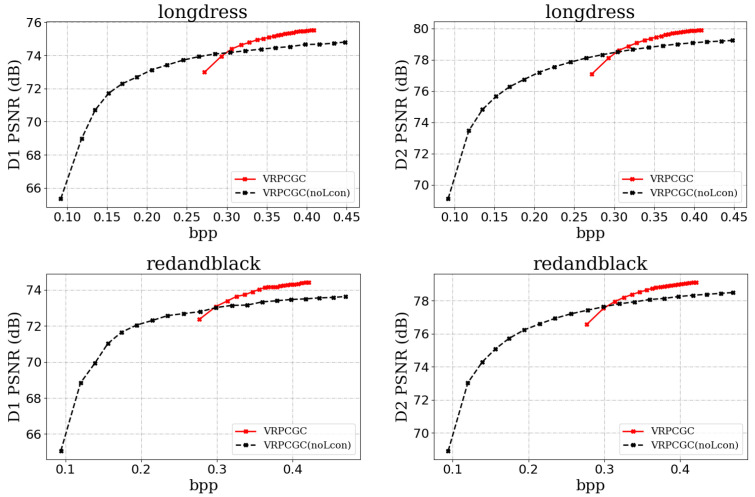
Visualization of the reconstructed point cloud, where VRPCGC denotes a variable rate model based on contrastive learning while VRPCGC(noLcon) denotes the model optimized only by the sum of the R-D pairs.

As shown in Figure 10, the bit rate range achieved by VRPCGC(noLcon) is narrow and the model concentrates in the high bit rate region. The density of sampling points at high bit rates is much higher than that at low bit rates. Overall, VRPCGC (noLcon) performs better at high bit rates, but the model performance drops sharply when the bit rate decreases to a certain range. Though the model with contrastive learning does not perform well at high bit rates, its bit rate range is larger, which is of more practical value.

We concluded that the sum of the R-D pairs is not suitable for the VRPCGC, which does not yield a ideal bit rate range. The bit rate similarity penalty, at the cost of reducing the quality of the reconstructed point cloud, has the ability to expand the bit rate range to meet the practical requirements.

### 5.3. Boundary Learning

We could set different ε to explore its impact on the model. We have exemplified our studies using *Longdress*. To simplify the experiment and facilitate comparison, this subsection analyzes the performance through the single rate model. The performance was evaluated using D1 PSNR in a fixed bit rate.

**Figure 11:**
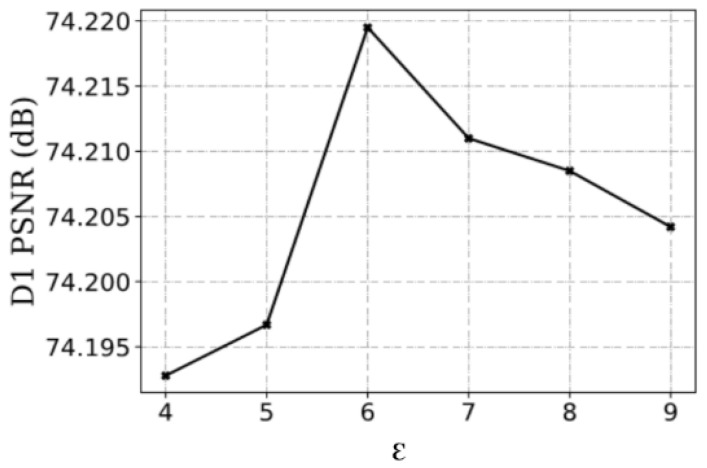
Visualization of the reconstructed point cloud.

As shown in Figure 11, the model performance increases and then decreases when gradually increasing. This is because when the number of boundary points is too large, the effect of boundary points is diluted, which degrades the quality of reconstructed point cloud. The peak occurs when ε=6.

**Figure 12:**
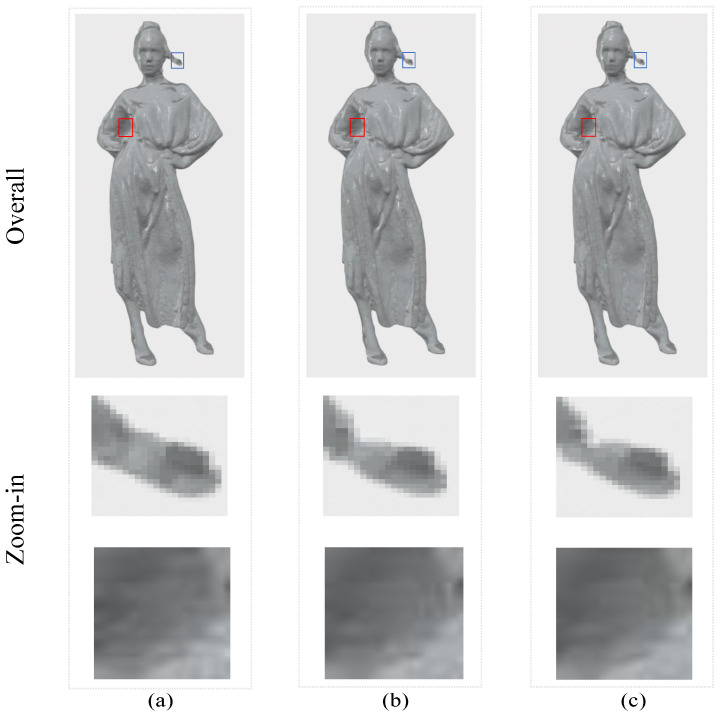
Visualization of the reconstructed point cloud (**a**–**c**).

Figure 12 shows the visualization of the reconstructed point cloud, and the detail part can be viewed by zooming. Figure 12a is the ground-truth, which is used as a reference comparison. Figure 12b shows the visualization of the reconstructed point cloud with the boundary learning added while Figure 12c without. The model classifies the boundary points better after adding boundary learning. For example, the hair in Figure 12b is more complete, while Figure 12 loses some points. Moreover, boundary learning increases the texture reconstruction ability of the model, which is due to the delineation of boundary points that makes the texture more clear.

## 6. Conclusions

We have proposed a variable rate point cloud compression algorithm, which solves the problem of single correspondence between model and bit rate of learned compression algorithm by adjusting the model bit rate through hyperparameters. The ScalingNet based on convolutional operations is proposed for feature modulation, which modulates the latent features according to the the Lagrange multipliers λ to obtain the model bit rate that meets the expectation. The geometric information of current point cloud is added to the ScalingNet, so that the scale network can learn the spatial information and achieve modulation of the features. Second, a bit rate expansion method based on contrastive learning is proposed for variable rate model. The model bit rate distribution is pulled apart by contrastive learning and bit rate range is improved. Finally, a point cloud reconstruction method based on boundary learning is proposed to achieve better visualization effects by focusing on boundary points. The experimental results demonstrate that the model achieves variable rate PCC with guaranteed performance. By training a single model, a large range of bit rate compression is achieved, which avoids the time cost caused by multiple model training and increases the practical application value of point cloud compression.

## Figures and Tables

**Table 1:** Test data format.

Point Cloud		Points	Precision
MVUB	Andrew	279,664	9 [t]
	Davaid	330,791	9
	Phil	356,256	9
	Sarah	302,437	9
8iVFB	Longdress	857,966	10
	Loot	805,285	10
	Redandblack	757,691	10
	Soldier	1,089,091	10
Owill	Basketball player	2,925,514	11
	Dancer	2,592,758	11
	Excise	2,591,718	11
	Model	2,458,429	11

**Table 2:** BD-Rate gains against G-PCC (Octree), G-PCC (Trisoup), V-PCC, Learned-PCGC, PCGCv2 in D1 and D2 based BD-Rate measurement. The ‘-’ means that BD-rate can’t be quantified by the area difference between the two curves due to the insufficient bit rate overlap.

Point Cloud		D1					D2				
		G-PCC (octree)	G-PCC (trisoup)	V-PCC	Learned-PCGC	PCGCv2	G-PCC (octree)	G-PCC (trisoup)	V-PCC	Learned- PCGC	PCGCv2
8iVFB	Longdress	−81.70	−62.32	26.39	−10.46	44.24	−79.16	−75.88	−0.84	−19.77	25.51
	Loot	−81.90	−67.10	20.41	−16.77	45.22	−80.04	−79.63	−4.82	−25.20	22.42
	Redandblack	−81.58	−64.34	−5.09	−11.88	36.69	−79.25	−74.44	−27.26	−20.94	22.39
	Soldier	−82.01	−64.20	5.59	−14.09	34.21	−79.43	−76.70	−17.12	−19.65	19.44
	average	**−81.80**	**−64.49**	**11.83**	**−13.30**	**40.09**	**−79.47**	**−76.66**	**−12.51**	**−21.39**	**22.44**
Owill	Basketball player	−84.56	-	50.79	−13.46	76.99	−84.51	-	17.74	−25.17	35.25
	Dancer	−84.93	-	50.12	−24.64	67.81	−84.47	-	17.29	−28.03	32.38
	exercise	−82.79	-	73.64	−28.41	70.04	−80.85	-	38.40	−26.17	38.67
	model	−85.02	-	62.97	−32.45	59.47	−78.67	-	27.57	−31.34	33.33
	average	**−84.33**	-	**59.38**	**−24.74**	**68.58**	**−82.13**	-	**25.25**	**−27.68**	**34.91**
Overall average		**−83.06**	**−64.49**	**35.60**	**−16.91**	**54.33**	**−80.80**	**−76.66**	**6.37**	**−24.53**	**28.67**

## Data Availability

The dataset used in this paper is the publicly available shapenet dataset, 8iVFB and Owlii. They can be downloaded at the following links: http://shapenet.cs.stanford.edu/shapenet/obj-zip and https://mpeg-pcc.org/index.php/pcc-content-database/ (accessed on 8 May 2023).
